# Lineage specification into GABAergic, glutamatergic, dopaminergic, and astrocytic phenotypes using MUSE stem cells: a novel approach for modeling neurodegenerative and psychiatric disorders

**DOI:** 10.1038/s41380-025-03251-2

**Published:** 2025-09-22

**Authors:** Domenico Aprile, Deanira Patrone, Sura Hilal Ahmed Al Sammarraie, Nicola Alessio, Gianfranco Peluso, Giovanni Di Bernardo, Umberto Galderisi

**Affiliations:** 1 Department of Experimental Medicine, Luigi Vanvitelli Campania University, Naples, Italy; 2 Genome and Stem Cell Center (GENKÖK) Erciyes University, Kayseri, Turkey; 3 University of Samarra, College of applied sciences, Department of Biotechnology, Samarra, Iraq; 4 International Medical University UNICAMILLUS, Rome, Italy; 5 Sbarro Institute for Cancer Research and Molecular Medicine, Center for Biotechnology, Temple University, Philadelphia, PA USA

**Keywords:** Stem cells, Molecular biology, Cell biology

## Abstract

The study of neurodegenerative and psychiatric disorders is often hampered by the limited accessibility of relevant neural tissues and the limitations of existing in vitro models. MUSE cells (Multilineage differentiating stress enduring), which are non-tumorigenic and stress-resistant stem cells, offer a robust alternative to traditional models such as induced pluripotent stem cells (iPSCs), which suffer from genetic variability and residual epigenetic memory. Possessing key pluripotency markers such as NANOG, OCT3/4, and SOX2, and capable of differentiating into all three germ layers, MUSE cells are ideally suited for both research and therapeutic applications. In this study, we have developed protocols for differentiating MUSE cells into neural progenitors, providing a critical foundation for modeling early neural development and dysfunction. These neural progenitors were then directed to specify into GABAergic, glutamatergic, dopaminergic neurons, and astrocytes, enabling detailed studies of specific lineage dysfunctions associated with neurodegenerative and psychiatric conditions such as schizophrenia, bipolar disorder, and Alzheimer’s disease. This approach not only enhances the physiological relevance of our models but also allows us to investigate the cellular mechanisms underlying these complex diseases more effectively. By improving our understanding of neural lineage specification and early developmental alterations, MUSE cells facilitate the development of targeted therapies and reduce reliance on animal models, thus advancing the path from research to clinical applications.

## Background

Neurological disorders often arise from dysfunctions within neuronal networks involving GABAergic, glutamatergic, and dopaminergic lineage specification, which are crucial for regulating neural excitation, mood, and behavioral responses [1]. Imbalances in these neurotransmitters can lead to conditions like schizophrenia, bipolar disorder, and depression, manifesting as both cognitive and behavioral abnormalities. For example, diminished GABA activity can increase neural excitability and anxiety, while excessive glutamatergic activity may induce excitotoxicity, contributing to mood instability seen in bipolar disorder [2–4]. Similarly, dopaminergic dysfunction is pivotal in various psychiatric disorders, influencing symptoms ranging from depression to schizophrenia [5–8]. Moreover, astrocytes play a vital role in maintaining neural homeostasis but undergo significant changes during neurodegenerative diseases, contributing to disease progression through mechanisms like reactive gliosis and impaired support of the blood-brain barrier [9, 10].

Many studies on neurological and psychiatric diseases focus on the dysfunction of mature neurons, while in vitro research aims to differentiate induced pluripotent stem cells (iPSC) into neurons to compare the functionality of patient-derived and healthy neurons. However, these studies face two major limitations. First, in vitro differentiation of neural precursor cells can only partially replicate neural activity, as in vivo neurons function within complex networks that cannot be fully reproduced in vitro. This limitation is particularly relevant when studying neurotransmitter release and neural circuit activation/deactivation, which require appropriate upstream stimuli and target connections. Second, many studies overlook critical biological processes occurring during neurogenesis.

Neural stem cells (NSCs) and neural progenitor cells (NPCs) play critical roles in brain development, plasticity and repair. However, alterations in their behavior and characteristics can lead to various neural pathologies [11]. Research indicates that the dysfunction of mature neurons in psychiatric disorders (e.g., bipolar disorder, schizophrenia) and neurological conditions (e.g., neurodegenerative and developmental disorders) is often linked to impaired neural stem cell activity [12]. These impairments include defects in cell commitment, lineage specification biases, and increased rates of premature cell death or senescence [13, 14]. Therefore, in vitro studies should adapt their objectives to address both the network-dependent nature of neural activity and the alterations in early neurogenesis that contribute to disease pathology. Many studies have overlooked this latter aspect, necessitating the development of new experimental approaches to bridge this scientific gap.

In this scenario, in vitro models using NSCs and NPCs are crucial for investigating neural pathological processes, as they allow for the study of molecular pathways involved in stem cell dysfunction and provide platforms for testing interventions aimed at restoring their normal function [15, 16]. Nevertheless, access to human biopsies for establishing NSC cultures remains limited [17–19]. This limitation has prompted researchers to explore alternative in vitro models for studying neurodegenerative and psychiatric disorders. Current models, including iPSCs, though promising, present challenges such as genetic variability and residual epigenetic memory, which can result in unreliable models [20–23]. This variability, combined with the critical role of epigenetic factors in disease onset and progression [24–27], underscores the necessity for more physiologically relevant in vitro models that can accurately replicate the cellular environments and mechanisms underlying these complex diseases [28].

In this context, MUSE cells (Multilineage differentiating stress enduring), a non-tumorigenic, stress-resistant population of stem cells discovered in bone marrow and other tissues like connective tissue and peripheral blood, offer unique advantages [29]. MUSE cells express key pluripotency markers such as *NANOG*, *OCT3/4*, and *SOX2*, and are capable of differentiating into all three germ layer derivatives—endoderm, mesoderm, and ectoderm—both spontaneously and under specific induction conditions. Additionally, their ability to home to damaged tissues and avoid immune rejection makes them particularly well-suited for therapeutic applications and regenerative medicine [30–34].

In this study, we specifically focused on NPCs as a foundational model for dissecting the early stages of neural differentiation. Our objective was to develop efficient protocols to study MUSE cells lineage specification into critical neural cell types—GABAergic, glutamatergic, dopaminergic neurons, and astrocytes. This focus on NPCs allows us to explore how early neural fate decisions contribute to the development of these distinct neuronal and glial lineages, which play pivotal roles in both normal brain function and the pathogenesis of neurodegenerative and psychiatric disorders. By establishing detailed differentiation protocols for these lineages from MUSE-derived NPCs, we provide a valuable framework for studying the cellular dynamics that underpin neural development and disease.

By utilizing MUSE stem cells, we have established robust in vitro models that enhance our understanding of neurological and psychiatric disorders. These models facilitate the development of targeted therapies and reduce reliance on animal testing, promoting an ethical and efficient approach to advancing medical research. This underscores the potential of in vitro models to transform the study and treatment of brain diseases.

## Materials

### Equipment

Burker cell counter (Sigma, cat. n. Z359629)

Centrifuge (Thermo Scientific, model Heraeus Megafuge 1.0)

Coverslip (Sigma, cat. n. Z375357)

Dry ice

Falcon conical tube, polypropylene, centrifuge tubes (50 ml; Falcon, cat. no. 100-0090, 38010)

Falcon conical tubes, polypropylene, centrifuge tubes (15 ml; Falcon, cat. no. 100-0092, 38009)

Filter (0.22 µm Millipore, cat. no. SAMP2GPNK)

Filtered pipette tips (10 μl; BioPoint Scientific, cat. no. 311-4050)

Filtered pipette tips (20 μl; BioPoint Scientific, cat. no. 341-4050)

Filtered pipette tips (200 μl; BioPoint Scientific, cat. no. 351-4050)

Flow Cytometer (Millipore, model Guava® easyCyte 5 HPL Benchtop Flow Cytometer).

Humidified 95% O2/5% CO2 water jacketed incubator, 37 °C (Thermo Scientific, model Forma Series II)

Inverted microscope (Leica, DMIL 090-135.001)

Leica DM2000 fluorescence microscope and DMC5400 camera (Leica)

Line-Gene 9600 system (Bioer Technology)

Low-binding culture dishes (100 mm, Corning, cat. no. 4615)

Microscope slides (75 mm × 25 mm; Corning, cat. no. CLS294875X25)

MidiMACS™ Separator and Starting Kits (Miltenyi biotec, cat. no 130-042-301)

MS Columns (Miltenyi biotec, cat. no.130-042-201)

Multidish (24 well; Falcon, cat. no. 353047)

NanoDrop One Microvolume UV-Vis Spectrophotometers (Thermo Fisher, cat. no ND-ONE-W)

Pipette (1,000 μl; Gilson, cat. no. MSF123602)

Pipette (2 μl; Gilson, cat. no. MSF144801)

Pipette (20 μl; Gilson, cat. no. MSF123600)

Pipette (200 μl; Gilson, cat. no. MSF123601)

Round coverslip (15 mm; Labmaterial, cat. no. 550010-C)

Serological disposable pipette (10 ml; BD Falcon, cat. no. 357551)

Serological disposable pipette (25 ml; BD Falcon, cat. no. 357525)

Serological disposable pipette (5 ml; BD Falcon, cat. no. 357543)

Tissue culture dish (100 mm; Falcon, cat. no. 353003)

Tissue culture dish (150 mm; Falcon, cat. no. 353025)

Tissue culture dish (60 mm; Falcon, cat. no. 353002)

### Reagents

5X ALL-IN-ONE RT MasterMix (ABM, cat. no. G592)

Accutase 1X (Sigma, cat. no. SCR005)

Adenosine 3′,5′-cyclic Monophosphate, N6,O2′-Dibutyryl-, sodium salt (cAMP, cat. no. 28745-M)

anti-FITC microbeads (Miltenyi biotec, cat. no. 130-048-701)

Antibiotic Solution 100× Liquid, 10,000 U Penicillin and 10 mg Streptomycin per ml in 0.9% normal saline (Himedia, cat. no. A001-100ML)

Antibodies for immunocytochemical analysis (Table 1)

**Table 1:** Primary and secondary antibodies.

Antigen	Primary or secondary	Company	Cat. no	Dilution	Application
OCT3/4	Pr	ElabScience	E-AB-36156	1:200	Pluripotency marker
SOX2	Pr	ElabScience	E-AB63542	1:200	Pluripotency marker
NANOG	Pr	ElabScience	E-AB-70199	1:300	Pluripotency marker
SSEA3	Pr	Biolegend	330302	1:50	Pluripotency marker
TUBB3	Pr	ElabScience	E-AB-20033	1:200	Neuronal (ectodermal) marker
MAP2	Pr	AbCam	ab5392	1:500	Neural marker
NESTIN	Pr	ElabScience	E-AB-63599	1:200	Neuronal progentor marker
GABA	Pr	AbCam	ab86186	1:500	Gabaergic nueron marker
VGluT1	Pr	AbCam	ab77822	1:300	Glutamatergic neuron marker
GLS	Pr	ElabScience	E-AB-19340	1:200	Glutamatergic neuron marker
TH	Pr	Sigma	AB1542	1:400	Dopaminergic neuron marker
GFAP	Pr	ElabScience	E-AB-70040	1:200	Classic astrocyte marker
S100-B	Pr	ElabScience	E-AB-10118L	1:200	Early Astrocyte Marker
FITC anti-rat IgM	Sc	Biolegend	408905	1:500	Muse cells Marker
Goat anti-rabbit IgG, DyLight 488 conjugate	Sc	Thermo-Fisher	35552	1:400	Secondary antibody
Goat anti-Mouse IgG, DyLight 594 Conjugate	Sc	Thermo-Fisher	35510	1:400	Secondary antibody

Ascorbic Acid 2- Phosphate, 100 mM (Millipore, cat. no 2004011)

Astrocyte Growth Medium (Sigma, cat. no. 821-500)

Astrocyte Growth Supplement (Sigma, cat. no. 821-GS)

B-27™ Plus Supplement (50X) (Gibco, cat. no A3582801)

Brain-derived neurotrophic factor, Human Recombinant Protein (BDNF; PeproTech, cat. no. 450-02)

BrainPhys^TM^ Neuronal Medium (Stem Cell Technologies, cat. 05790)

BrightGreen 2X qPCR MasterMix (ABM, cat. no. G891)

BSA (Gibco, cat. no.16140071)

CHIR-99021- GSK-3 inhibitor (Sigma-Aldrich, cat. no. SML1046-5MG)

DAPI (Thermo Fisher, cat. no. 62248)

DMSO (Sigma, cat no. C6164)

Dulbecco’s modified Eagle medium, high glucose 1× (DMEM; Invitrogen, cat. no. 11965-092)

Dulbecco’s Phosphate buffered saline D-PBS (Sigma, cat. no. D8662)

EDTA (Millipore, cat. no 324503)

Epidermal growth factor (EGF; PeproTech, cat. no. GMP10015)

Fetal Bovine Serum South America Origin, Low Endotoxin level < 0,3 EU/ml (FBS; Microgem, cat. no. RM10432-500ML)

Fibroblast Growth Factor-8 Human Recombinant (FGF-8; Millipore, cat. no. 617103)

FluoroBrite DMEM (Gibco, cat. no. A18967-01)

Formaldehyde solution 37% (Sigma-Aldrich, cat. no. F8775)

Glial-Derived Neurotrophic factor, Human Recombinant Protein (GDNF; PeproTech, cat. no. 45010)

KnockOutTM DMEM/F-12 (Thermo Fisher, cat. no.12660012)

L-Glutamine (Gibco, cat. no. 25030081)

Laminin (Sigma, cat no. L2020)

Matrigel® (Corning, cat. no. 354277)

Methylcellulose MethoCult H4100 (StemCell Technologies, cat. no. 04100)

N-2 Supplement (100×) (Gibco, cat. no 17502048)

Neurobasal™ Medium (Gibco, cat. no. 21103049)

Neurotrophic Factor Human Recombinant Transforming Growth Factor bIII (TGF-b-III; Millipore, cat. no. 617106)

Noggin (Sigma, cat. no. SRP4675)

Non-Essential Amino Acid (NEAA) (Gibco, cat no. 11140050)

Poly (2-hydroxethyl methacrylate) (poly-HEMA; Sigma, cat. no. P3932)

Poly-l-ornithine solution (0.1% (wt/vol) in H2O; Sigma, cat. no. P4957-50ML)

Primer sequences for RT-PCR (Table 2)

**Table 2:** Primer information for RT-qPCR.

Gene	Primer sequences	T^a^	Size	Application
*SOX2*	5′ - ACAACTCGGAGATCAGCAAGC- 3′ 5′- TCATGAGCGTCTTGGTTTTCC - 3′	60	139	Pluripotency marker
*NES*	5′ - TCTTGACCAGGAGATAGC - 3′ 5′ - CGCAGACTTCAGTGATTC - 3′	56	127	Neural stem cell marker
*TUBB3*	5′- AGATGGAGATGATGAAGAT – 3′ 5′- TATCAACTAATACGGAGATT – 3′	56	112	Neuronal marker
*NEFH*	5′ - TCAAGGATCAGAGTAACAC – 3′ 5′ - GCTTAACATATTGCTAACAGAA –3'	55	112	Neuronal marker
*MAP2*	5′- TCAGAGTATGTCAGTCCAA - 3′ 5′ - ACACGAGTCCATCCTAAT - 3′	55	132	Neuronal marker
*GAD1*	5′- CCATGGATGCACCAGAAAACT – 3′ 5′- AGGATTGCCTCTCCTTGAAGG – 3'	60	109	Gabaergic nueron marker
*GAD2*	5′- TACTCTTCAGAATATGGAC – 3′ 5′ - TTGGCACACCTAACTAA - 3′	57	145	Gabaergic nueron marker
*GLS*	5′ - GCTGGTAATGAATATGTTG – 3′ 5′ - TGTCTGTGCCTTCTGGAA – 3'	55	123	Glutamatergic neuron marker
*VGLUT1*	5′ - TATAGAGAAGTCACAGAAT – 3′ 5′ - GATTTACAGTCACAGAGA – 3'	56	107	Glutamatergic neuron marker
*DAT*	5′- CCACGTTCAAAGTACTCGGCA – 3′ 5′- TGCAACAACTCCTGGAACAGC – 3'	61	121	Dopaminergic neuron marker
*TH*	5′- AGTCTACTTCGTGTCTGAG – 3′ 5′ - GTCGAACTTCACGGAGAA – 3'	60	94	Dopaminergic neuron marker
*SOX9*	5′- CCGTCACCATGAGCCAGG – 3′ 5′ - AGAGGGTCTCTCGTCTTTAG3 – 3′	59	126	Progenitor marker
*S100B*	5′-CCTCATCGACGTTTTCCACC -3′ 5′- TCCACAACCTCCTGCTCTTT – 3′	56	112	Astrocyte marker
*APOE*	5′- GCCTCAAGAGCTGGTTCGA - 3′ 5′ – TTCGGCGTTCAGTGATTGTC – 3′	55	129	Astrocyte marker
*SLC1A2*	5′ – CAGTTATAGGACACAGTT – 3′ 5′ – TTCGGCGTTCAGTGATTGTC – 3′	54	146	Astrocyte marker
*GFAP*	5′ - GCAGACCTTCTCCAACCT – 3′ 5′ - GTATAACTCGTATTGTGAGGCTT – 3'	58	103	Astrocyte marker
*GAPDH*	5′ - GGAGTCAACGGATTTGGTCGT - 3′ 5′ - ACGGTGCCATGGAATTTGC - 3′	58	160	Housekeeping

T^a^= annealing temperature.

Recombinant Human FGF2-basic (bFGF2; PeproTech, cat. no. 100-18B5-0UG)

RNeasy Mini Kit (Qiagen, cat. No. 74104)

SB-431542, TGF-β Receptor Kinase Inhibitor (Med Chem Express, cat. no. HY-10431)

Sonic HedgeHog, Human Recombinant (Shh; Millipore, cat. no. 617102)

Triton X-100 (Sigma-Aldrich, cat. no. X100)

Trypan blue (Sigma Aldrich, St Louis, MO, USA, cat. no. T8154).

Trypsin-EDTA solution (0.25% (wt/vol) trypsin/1 mM EDTA-4Na (1X), liquid; Invitrogen, cat. no. 25200-072)

Water, double distilled (DDW)

Y-27632 10 mM (Sigma, cat. no. 688002)

### Procedure

#### Isolation and characterization of Muse cells from skin stromal cells

**Fig. 1:**
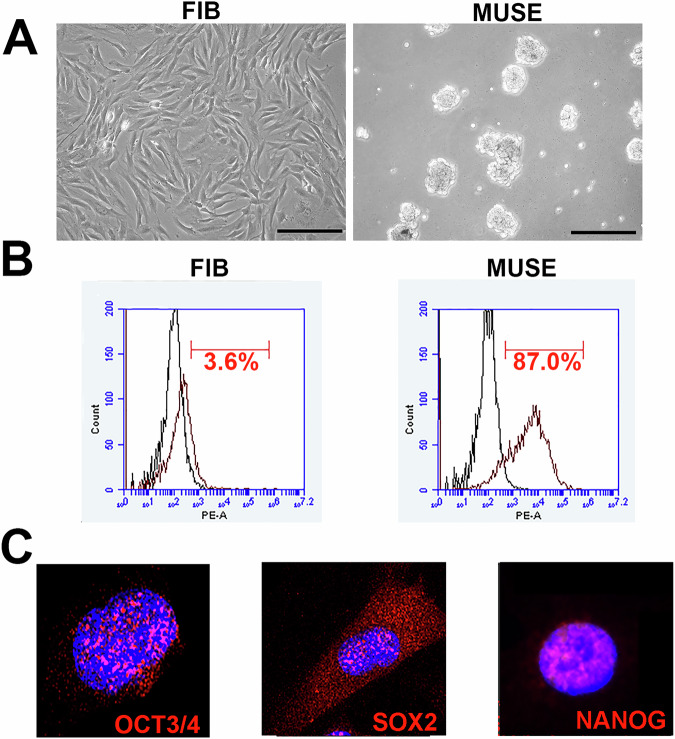
In vitro characterization of MUSE cells. **A** The picture shows a representative image of stromal and fibroblast cells (FIB) and MUSE cells visualized through an inverted microscope (Leica DMIL 090-135.001). 20X magnification. Scale bar 100 µm. **B** Expression of SSEA-3 surface markers measured by flow cytometry in FIB and MUSE cells. **C** The pictures are representative images of immunocytochemistry for stemness markers: OCT3/4, SOX2 and NANOG, performed on MUSE cells. The nuclei were counterstained with DAPI (blue). 20X magnification.

The experimental procedures adhered to the guidelines approved by the Ethics Committee of the University of Campania Luigi Vanvitelli (protocol no. 0013954/i). All participants were informed about the nature of the research and provided consent for the use of their biological samples. Skin biopsies were performed using a 1.5 mm punch under medical supervision. The biopsies were processed in vitro to isolate and expand stromal cells. Skin biopsy samples were collected from five donors, aged 25–40 years. Confluent stromal cells and fibroblasts (Fig. 1A) at passage 4 (P4) were harvested using 0.25% trypsin–EDTA and subjected to cell sorting to isolate MUSE cells. The cells were suspended in MACS buffer, which contained 0.5% bovine serum albumin (BSA) and 2 mM EDTA-2H2O in FluoroBrite DMEM. They were then incubated with an anti-human SSEA-3 antibody for 1 h on ice. After this, the cells were washed three times with MACS buffer and centrifuged at 400 g for 5 min. The cells were next incubated with a secondary antibody, anti-rat IgM-FITC, for 1 h on ice and washed three more times. Following that, the cells were incubated with anti-FITC microbeads for 15 min on ice and then washed again with MACS buffer. Magnetic-activated cell sorting (MACS) was used to isolate SSEA-3(+) cells, representing MUSE cells, and SSEA-3(−) non-MUSE cells, according to the manufacturer’s instructions (Miltenyi). The collected SSEA-3 positive cells, which represented MUSE cells, were then cultivated in suspension for 7 days in poly-2-hydroxyethyl methacrylate (pHEMA)-coated petri dishes with a complete medium. The medium consisted of DMEM low glucose, supplemented with 10% FBS, 4 mM L-glutamine, 50 U/ml penicillin–streptomycin, and 2.6% MethoCult [35].

MUSE cells were cultured on pHEMA for 7 days to select and enrich the population of MUSE stem cells (Fig. 1A). When grown in suspension, MUSE cells overexpress stemness markers such as OCT3/4, SOX2, and NANOG. At the end of the 7 days, the MUSE cells were characterized for the expression of the surface marker SSEA-3 using flow cytometry and for the expression of stemness markers using immunocytochemistry (Fig. 1B, C).

MUSE cells were washed with PBS and incubated with anti-SSEA-3 PE-conjugated antibody, following the manufacturer’s instructions. After 30 min of incubation with the antibodies in the dark at room temperature, the cells were washed again with PBS and resuspended in MACS buffer for analysis using a Guava easyCyte flow cytometer. Data analysis was performed following standard procedures using easyCyte software, with at least 5000 cells per sample analyzed, and gating was done based on forward scatter (FSC) and side scatter (SSC) channel signals (Fig. 1B).

 cells were seeded on coverslips in a 24-well plate overnight and then fixed with a 4% formaldehyde solution for 15 min at room temperature. After fixation, the cells were washed three times with PBS and permeabilized with 0.3% Triton X-100 in PBS for 15 min. After another PBS wash, the cells were incubated for 1 h at room temperature in a blocking solution consisting of 0.1% Triton X-100 and 5% bovine serum in PBS. The cells were then incubated with primary antibodies (OCT3/4, SOX2, NANOG; see Table 1) in blocking solution overnight at 4 °C, following the manufacturer’s instructions for each antibody. The next day, after three washes with PBS (5 min each), the cells were incubated with fluorophore-conjugated secondary antibodies for 1 h at room temperature in the dark. After three more PBS washes (5 min each), the coverslips were mounted. Nuclear staining was performed using a DAPI mounting medium, and micrographs were taken using a fluorescence microscope (Leica)(Fig. 1C).

#### Neural progenitor differentiation

##### Preparation of Matrigel-coated plates

Matrigel is used to pre-coat cell culture dishes and provide an attachment surface for feeder-free human cells to replicate on. To avoid warming up Matrigel stock and solidifies at temperatures above 10 °C, special care is required during its preparation.

1. **Defrost Matrigel**: 1 day before use, defrost the matrigel at 4 °C overnight on ice or in a cold chamber to maintain the temperature during the whole process.
2. **Prepare the coating solution**: Dilute Matrigel 1× at a ratio of (1:50) using pre-cooled DMEM/F12 in a Falcon tube and transfer the desired volume to the plate. Add 5 mL in 100 mm plates and 2 mL in 60 mm plates. Gently shake the mix with a pipette, avoiding the formation of bubbles. Next, distribute it evenly on each culture plate. If necessary, gently tap the plate on the work surface to make sure the liquid covers the entire surface evenly. Incubate the Matrigel-coated plates at 37 °C and 5% CO₂ for at least 1 h.
3. **Remove the solution**: After incubation, aspirate excess liquid Matrigel from the plates and immediately add cells or culture medium to the plate to prevent the Matrigel coating from drying out.
4. **Preparation before use**: Just before use, aspirate the solution from the coated cultureware and add the Neuronal Differentiation Medium to the cultureware.

#### Induction of Neural differentiation (day 1 to 13)

MUSE cells grown in suspension on pHEMA for 7 days are collected using a pipette into a 15 ml Falcon tube. The plate is washed thoroughly with PBS to collect all the cells, and the liquid is added to the 15 ml Falcon tube. Subsequently, the cells are centrifuged for 5 min at 300 g at room temperature. The supernatant is discarded, and the pellet is resuspended in 1 mL of Neural Induction Medium 1 (NIM1: DMEM/F12, 50 U/ml penicillin–streptomycin, N2 1%, B27 4%, L-Glut 1 mM, BSA 1 mg/mL, NEAA 1%, SB431542 TGF-β receptor inhibitor 10 µM, Noggin 200 ng/mL, Laminin 1 µg/mL, Table 3). The cells are seed on Matrigel coated dish, subsequently other 7 ml of NIM1 are added to the dish. The cells are then collected and plated at a density of 1 × 10^5^ on a 100 mm Corning plate coated with Matrigel, which has been prepared in advance. After plating the cells on Matrigel, an additional 8 mL of fresh NIM1 is added. Half of the medium, approximately 4 mL, is removed and replaced with freshly prepared NIM1 every 3 days until day 7.

**Table 3:** Media composition for a final volume of 10 ml.

Neural Induction Medium 1	Stock concentration	Final concentration	Volume 10 mL
DMEM-F12			8.998 mL
N2	100%	1%	100 µL
B27	100%	4%	400 µL
BSA	5%	0.1%	200 µL
SB431542	10 mM	2 µM	2 µL
Noggin	20 µg/mL	200 ng/mL	100 µL
NEAA	100%	1%	100 µL
L-GLUT	200 mM	1 mM	50 µL
Pen-strep	10.000 U/µL	50 U/µL	50 µL

Neural Induction Medium 2	Stock concentration	Final concentration	Volume 10 mL
DMEM-F12			9.080 mL
N2	100%	1%	100 µL
B27	100%	4%	400 µL
BSA	5%	0.1%	200 µL
NEAA	100%	1%	100 µL
Laminin	500 µg/mL	1 µg/mL	20 µL
L-GLUT	200 mM	1 mM	50 µL
Pen-strep	10.000 U/mL	50 U/µL	50 µL

Neural Progenitor Medium	Stock concentration	Final concentration	Volume 10 mL
DMEM-F12			9.369,5 mL
N2	100%	1%	100 µL
B27	100%	4%	400 µL
EGF	100 µg/mL	20 ng/mL	4 µL
FGF2	50 µg/mL	20 ng/ mL	4 µL
Rock inhibitor	10 mM	2,5 µM	2,5 µL
Laminin	500 µg/mL	1 µg/ mL	20 µL
L-GLUT	200 mM	1 mM	50 µL
Pen-strep	10.000 U/mL	50 U/µL	50 µL

Neuronal Differentiation Medium	Stock concentration	Final concentration	Volume 10 mL
BrainPhys Medium			9.426
N2	100%	1%	100 µL
B27	100%	4%	400 µL
Laminin	500 µg/mL	1 µg/ mL	20 µL
BDNF	100 µg/ mL	20 ng/ mL	2 µL
GDNF	100 µg/ mL	20 ng/ mL	2 µL
Pen-strep	10.000 U/mL	50 U/µL	50 µL

Astrocyte Differentiation Medium	Stock concentration	Final concentration	Volume 10 mL
Astrocyte Growth Medium			9.550 mL
Astrocyte Growth Supplement	100%	2%	200 µL
FBS	100%	2%	200 µL
Pen-strep	10.000 U/mL	50 U/µL	50 µL

Dopaminergic Induction Medium 1	Stock concentration	Final concentration	Volume 10 mL
DMEM-F12			9.640 mL
N2	100%	1%	100 µL
SB431542	10 mM	10 µM	10 µL
Noggin	20 µg/mL	200 ng/mL	100 µL
L-GLUT	200 mM	2 mM	100 µL
Pen-strep	10.000 U/µL	50 U/µL	50 µL

Dopaminergic Induction Medium 2	Stock concentration	Final concentration	Volume 10 mL
DMEM-F12			8,888 mL
N2	100%	1%	100 µL
B27	100%	4%	400 µL
SB431542	10 mM	10 µM	10 µL
Noggin	20 µg/mL	100 ng/mL	50 µL
CHIR99021	4,3 mM	3 µM	7 µL
SHH	5 µg/mL	100 ng/mL	200 µL
FGF8	5 µg/mL	100 ng/mL	200 µL
L-GLUT	200 mM	2 mM	100 µL
Pen-strep	10.000 U/µL	50 U/µL	50 µL

Dopaminergic Induction Medium 3	Stock concentration	Final concentration	Volume 10 mL
Neurobasal			8.968 mL
N2	100%	1%	100 µL
B27	100%	4%	400 µL
Noggin	20 µg/mL	100 ng/mL	50 µL
CHIR99021	4,3 mM	3 µM	7 µL
SHH	5 µg/mL	100 ng/mL	200 µL
FGF8	5 µg/mL	100 ng/mL	200 µL
Laminin	500 µg/mL	1 µg/mL	20 µL
Pen-strep	10.000 U/µL	50 U/µL	50 µL

Dopaminergic Differentiation Medium	Stock concentration	Final concentration	Volume 10 mL
BrainPhys			9.791 mL
N2	100%	1%	100 µL
BDNF	100 µg/ mL	20 ng/mL	2 µL
GDNF	100 µg/ mL	20 ng/mL	2 µL
TGF-βIII	2 µg/ mL	1 ng/mL	5 µL
Ascorbic acid	100 mM	0.2 mM	20 µL
cAMP	0.5 M	0.5 mM	10 µL
Laminin	500 µg/mL	1 µg/mL	20 µL
Pen-strep	10.000 U/µL	50 U/µL	50 µL

**Fig. 2:**
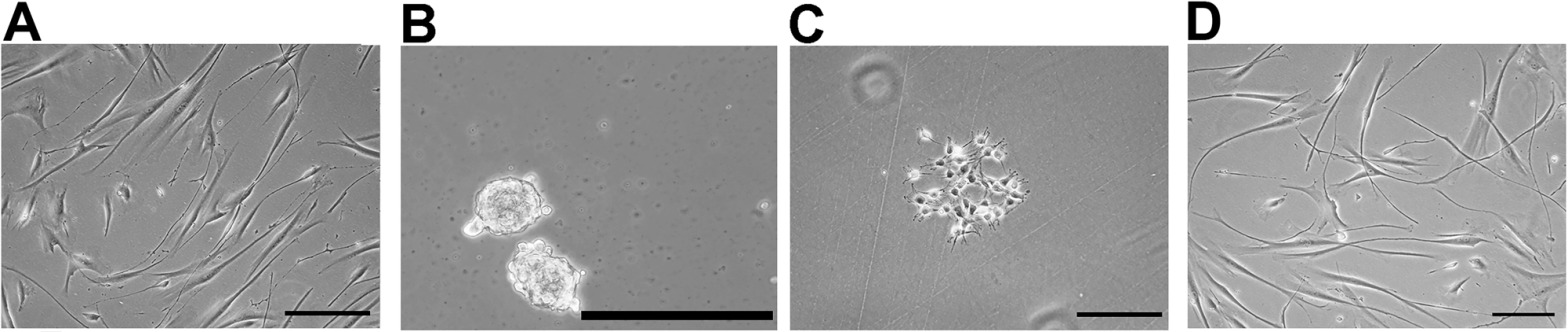
Morphological progression of MUSE cells toward neural progenitors. **A** Neural progenitor cells on day 13. **B** Neurosphere on day 16 on low-binding plates **C** Neurosphere adhering to Matrigel-coated plate. **D** Neuroprogenitor cells on day 30. Cells are visualized through an inverted microscope (Leica DMIL 090-135.001). 20X Magnification. Scale bar 100 µm.

During the first week, the cells exhibit proliferative capacity and can reach 80-90% confluence. Upon reaching day 7, the NIM1 medium is completely removed and replaced with Neural Induction Medium 2 (NIM2: DMEM/F12, 50 U/ml penicillin–streptomycin, N2 1%, B27 4%, L-Glut 1 mM, BSA 1 mg/mL, NEAA 1%, Laminin 1 µg/mL, Table 3) for 5 days. The medium is completely changed every day to ensure the removal of any inhibitors present in NIM1, until day 13 is reached. At this point, the cells should have reached 80–90% confluence (Fig. 2A).

#### Neurosphere formation and neural progenitor propagation (day 13 to 30)

On day 13, the cells have reached the first stage of neural differentiation, adopting a more elongated and branched morphology. The NIM2 medium is completely removed, and the plates are washed 3 times with PBS to thoroughly remove the medium. The cells are treated with Accutase 1× (3 ml for 100 mm plate) for 5 min at 37 °C in 5% CO₂. After the treatment, the cells detach from the plastic and are suspended in the petri dish. The cells are collected with 2 ml of FBS using a pipette to ensure that all the cells are gathered and then placed into a 15 ml Falcon tube. Subsequently, the cells are centrifuged for 5 min at 300 g at room temperature, the supernatant is removed, and the cells are resuspended in 1 ml of Neural Progenitor Medium (NPM: DMEM/F12, 50 U/ml penicillin–streptomycin, N2 1%, B27 4%, L-Glut 1 mM, Laminin 1 µg/mL, EGF 20 ng/mL, FGF2 20 ng/mL, Y-27632 ROCK inhibitor 1 µM, Table 3). The cells are plated on low-binding 100 mm plates at a density of 1 × 10⁶ for 3 days. After plating the cells on low binding plates, an additional 8 mL of fresh NPM is added.

After 3 days of growth in suspension, the cells form neurospheres (Fig. 2B). The cells create small clusters composed of multiple cells, which can later be collected and differentiated. At the end of the three days in suspension, the cells are collected with a pipette and placed into a 15 ml Falcon tube. The cells are centrifuged for 5 min at 300 g at room temperature, the supernatant is discarded, and the pellet is resuspended in 1 ml of Neural Progenitor Medium (NPM). The cells are counted and plated at a density of 5 × 10^5^ cells on a plate coated with Matrigel, which has been prepared in advance following the previous protocol. An additional 7 ml of NPM is added, and the cells are incubated at 37 °C with 5% CO₂ in a humidified atmosphere (Fig. 2C).

For the neurospheres plated on Matrigel with neural progenitor medium (NPM), half of the medium (approximately 4 mL) will be replaced twice a week with fresh medium until the cells reach confluence. In about 2–4 weeks, the cells will reach confluence and the neural progenitor stage (Fig. 2D). The cells will exhibit the following characteristics: an elongated or fusiform shape with thin processes extending from the cell body, which may become more numerous and branched as the cells differentiate into neurons or glia; a compact cell body and dynamic morphology, acquiring a more complex and branched structure as differentiation progresses. At this point, the neural progenitors can be harvested and used in experiments to characterize expression markers and analyze biological features. The progenitors can also be harvested and cryopreserved in DMSO and serum for future characterization or experimentation. Finally, the progenitors can be differentiated into GABAergic and glutamatergic neurons or into astrocytes (see protocols below).

#### Neurosphere plating issues

After the formation of neurospheres, it is common to lose part of the cell population when replating, as some cells may not adhere to the Matrigel-coated plate after the suspension step. This can occur because the neurosphere clusters do not adhere well to the substrate and are unable to readapt immediately. To address this issue, it is important to pipette the neurosphere cell pellet carefully and gently after centrifugation to break up the clusters and obtain single cells in suspension. Use a P200 pipette and gently rest the pipette tip on the side of the Falcon tube, avoiding rapid pipetting that could damage the cells. Then, replate the cells evenly onto the new Matrigel-coated plate. It is also good practice to start with a sufficiently high number of cells to minimize excessive cell loss during this phase.

After collecting the cells from suspension growth and plating them on Matrigel, the cells must reach confluence before starting the final step of neural differentiation. For this reason, the cells should no longer be trypsinized or replated. At this stage of the protocol, it is advisable to carefully plan the experiments and analyses to be conducted at the end of the differentiation protocol. Therefore, the cells should be plated in sufficient numbers and on appropriate plates to carry out the subsequent experiments.

#### Use of inhibitor in Neural differention

In neural differentiation in vitro, the use of SMAD pathway inhibitors such as Noggin, Y-27632 and SB431542 is essential to direct stem cells toward neural differentiation. The SMAD pathway is mostly activated by signals belonging to the families of transforming growth factors (TGF-β) and bone morphogenetic proteins (BMPs), which play a crucial role in the control of cell fate, including processes such as proliferation, differentiation, and apoptosis [36].

In particular, Y-27632 is an inhibitor of Rho-associated protein kinase (ROCK), a kinase involved in regulating the cytoskeleton by modulating actin polymerization. Rock is used to enhance cell survival, facilitate neurite outgrowth, and stabilize cell adhesion during the transition to a neural lineage reducing the risk of apoptosis induced by cell detachment (known as anoikis), which often occurs during stem cell culture and differentiation [37].

#### GABAergic and Glutamatergic differentiation (day 30 to 50)

The medium is completely removed from the neural progenitors, and the cells are washed 3 times with PBS. The medium is then replaced with Neuronal Differentiation Medium (NDM: BrainPhys Medium, 50 U/ml penicillin–streptomycin, N2 1%, B27 4%, Laminin 1 µg/mL, BDNF 20 ng/mL, GDNF 20 ng/mL, Table 3).

The cells were cultured in NDM for 20 days to allow differentiation into GABAergic and Glutamatergic neuronal phenotypes. Every 3 days, half of the NDM was removed and replaced with freshly prepared medium to avoid degradation of the growth factors, which have a short half-life.

**Fig. 3:**
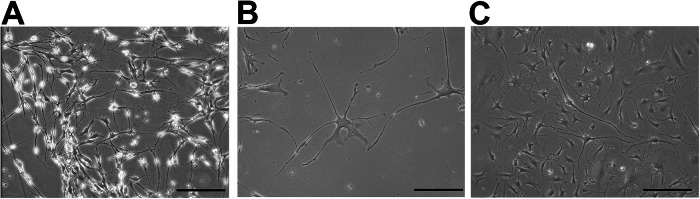
Differentiation of MUSE cells into neuronal and glial subtypes. **A** GABAergic and Glutamatergic differentiated cells. **B** Committed Astrocytes. **C** Dopaminergic differentiated cells. Cells are visualized through an inverted microscope (Leica DMIL 090-135.001). 20× Magnification. Scale bar 100 µm.

At the end of the 20 days, a portion of the mature neurons exhibited GABAergic or Glutamatergic phenotypes (Fig. 3A). GABAergic neurons are generally smaller and less branched compared to Glutamatergic neurons. They have a higher number of short, branched dendrites, often exhibiting complex dendritic branching. Glutamatergic neurons tend to have a more complex and branched morphology, with extended dendritic trees and a higher density of dendritic spines, corresponding to their role as the main excitatory neurons in the brain.

At the end of the differentiation process, the neurons can be collected for analysis of protein expression through immunocytochemistry or western blot, RNA expression analysis via RT-qPCR, or electrophysiological analysis of the neurons.

Neural committed cells were detached from the Matrigel-coated plates using Accutase 1× (3 ml for 100 mm plate) for 5–7 min at 37 °C with 5% CO₂. Once the cells were detached from the plastic, they were suspended in the petri dish. The cells were then collected with 2 ml of FBS using a pipette to ensure complete collection and transferred into a 15 ml Falcon tube. The cells were centrifuged at 300 g for 5 min at room temperature, and the supernatant was discarded. The cell pellet was resuspended in PBS to wash and remove any residual culture medium. Following another round of centrifugation, the pellet was resuspended in lysis buffer for RNA extraction. Alternatively, the cells can be resuspended in NDM and plated on poly-ornithine and laminin-coated plates for immunocytochemistry experiments.

#### Astrocyte differentiation (day 30 to 60)

The neural progenitors generated following the previously outlined protocol can also be used for astrocyte induction. After three washes with PBS to thoroughly remove all residual medium, 8 ml of Astrocyte Differentiation Medium (ADM: Astrocyte Growth Medium, Astrocyte Growth Supplement, 50 U/ml penicillin–streptomycin, FBS 2% fetal bovin serum, Table 3) are added to the neural progenitors for 30 days. During this period, half of the medium is replaced with fresh medium every three days. To avoid passaging or trypsinizing the cells, seed them at a low density. This comprehensive protocol enables the differentiation of MUSE cells into fully mature astrocytes. By the 30th day, a portion of the cells have developed a characteristic astrocytic phenotype (Fig. 3B). In vitro, these astrocytes typically display a star-shaped morphology with multiple branched processes extending from the cell body. These processes aid in synaptic support and the maintenance of homeostasis in neuronal environments. The cells exhibited also markers typical of mature astrocytes reflecting their functional and morphological maturity after the differentiation protocol.

Upon completion of the differentiation process, the astrocytes can be harvested for protein expression analysis using immunocytochemistry or western blot, or for RNA expression analysis through RT-qPCR (as detailed below).

#### Pre-dopaminergic neurons differentiation (day 1 to 14)

MUSE cells cultured in suspension on pHEMA for 7 days are collected with a pipette and transferred into a 15 mL Falcon tube. The plate is thoroughly washed with PBS to ensure all cells are collected, and the washing liquid is added to the same tube. The cells are then centrifuged at 300 g for 5 min at room temperature. After discarding the supernatant, the cell pellet is resuspended in 1 mL of Dopaminergic Induction Medium 1 (DIM1: DMEM-F12, pen-strep 50 U/mL, N2 1%, L-glutamine 2 mM, SB431542 (TGF-β inhibitor) 10 µM, Noggin 200 ng/mL, Table 3). The cells are plated onto a 100 mm dish coated with Matrigel (see protocol above), and an additional 7 mL of DIM1 is added. The cells are cultured in this medium for 5 days, with the medium being replaced with fresh DIM1 every two days. On the fifth day, the medium is removed, and the cells are washed twice with PBS to completely eliminate residual medium and any factors. Then, 8 mL of Dopaminergic Induction Medium 2 (DIM2: DMEM-F12, pen-strep 50 U/mL, N2 1%, B27 4%, L-glutamine 2 mM, SB431542 10 µM, Noggin 100 ng/mL, SHH 100 ng/mL, FGF8 100 ng/mL, CHIR99021 (GSK-3 inhibitor) 3 µM, Table 3) is added. The medium is replaced every two days with fresh DIM2.

On the tenth day, the medium is fully removed, and the cells are washed with PBS. Dopaminergic Induction Medium 3 (DIM3: Neurobasal Medium, pen-strep 50 U/mL, N2 1%, B27 4%, Laminin 1 µg/mL, Noggin 100 ng/mL, SHH 100 ng/mL, FGF8 100 ng/mL, CHIR99021 3 µM, Table 3) is added. The cells are cultured in this medium until day 14, with the medium being replaced every two days. During this phase, the cells will continue to proliferate, reaching the neural precursor stage with a predisposition toward differentiation into dopaminergic neurons.

#### Dopaminergic neurons differentiation (day 14 to 30)

##### Preparation of poly-L-ornithine and laminin-coated plates

Neural progenitor cells should be cultured on poly-L-ornithine and laminin-coated culture ware before starting terminal differentiation. The following steps are recommended:

1. Prepare the coating solution: Dilute the Poly-L-Ornithine solution 0.1 mg/mL (1:10) with PBS to achieve a final concentration of 10 µg/mL. Add enough volume of the Poly-L-Ornithine solution to fully cover the surface of the cultureware (5 mL in 100 plate). Incubate at room temperature for 2 h.
2. Prepare the Laminin: Thaw the laminin, provided at 1 mg/mL, on ice. Using sterile 1X PBS, dilute the laminin to a final concentration of 10 µg/mL.
3. Remove the Poly-L-Ornithine solution: Aspirate the Poly-L-Ornithine solution. Add sufficient volume of the 10 µg/mL laminin solution to cover the entire surface of the cultureware (5 mL in 100 plate). Incubate at room temperature for 2 h.
4. Storage of coated plates: Coated plates and flasks can be stored in the laminin solution at −20 °C for up to six months. Wrap the plates in plastic wrap before storing them at −20 °C.
5. Preparation before use: Just before use, aspirate the laminin solution from the coated cultureware and wash once with 1× PBS. Add the Neuronal Differentiation Medium to the cultureware. Do not allow the plates or wells to dry out, as this may result in uneven cell attachment.

On day 14, the cells are washed twice with PBS, and the medium is replaced with Dopaminergic Differentiation Medium (DDM: BrainPhys medium, pen-strep 50 U/mL, N2 1%, Laminin 1 µg/mL, BDNF 20 ng/mL, GDNF 20 ng/mL, TGF-βIII 1 ng/mL, ascorbic acid 0.2 mM, cAMP 0.5 mM, Table 3). The cells are cultured in this medium until reaching confluency (around day 20), with the medium being refreshed every 3 days. At confluency, on day 20, the cells are detached from the Matrigel-coated plates using Accutase (3 mL) for 5–7 min at 37 °C with 5% CO₂. Once detached, the cells are suspended in the Petri dish, collected in 1 mL of DDM, and counted. The cells are centrifuged at 300 g for 5 min and resuspended in the appropriate volume of DDM. Subsequently, the cells are seeded onto Poly-L-ornithine/Laminin-coated plates (see protocol above) at a desired concentration, depending on the planned experiments and analyses.

The neural cells are grown for approximately 10 days in DDM to allow for the final differentiation into dopaminergic neurons (Fig. 3C). The medium is replaced with fresh DDM every 3 days. At the end of differentiation, a portion of the cells will exhibit the phenotypic characteristics of mature neurons, such as: Morphological features: Dopaminergic neurons typically display a well-defined morphology, characterized by the presence of long, branched dendrites and a single axon. The dendritic arborization allows for extensive synaptic connections, facilitating communication with other neurons. Neurite outgrowth: Following differentiation, these neurons often extend long neurites that can branch extensively, which is crucial for their functional connectivity. Cell body shape: The cell bodies of dopaminergic neurons tend to be relatively larger and are often round or oval in shape, containing prominent nuclei.

Upon completion of the differentiation process, the dopaminergic neuron can be harvested or fixed for protein expression analysis using immunocytochemistry or western blot, or for RNA expression analysis through RT-qPCR (as detailed below).

#### Use of inhibitor and growth factor in dopomergic neuron differention

In dopaminergic differentiation, the use of specific inhibitors is essential to guide stem cells toward a neuronal fate and promote the formation of dopaminergic neurons. In particular, *SB431542* is a specific inhibitor of TGF-β (transforming growth factor beta) receptor kinases, specifically ALK4, ALK5 and ALK7 receptors. Inhibition of Activin/Nodal/TGF-β signaling pathways, which normally induce maintenance of the undifferentiated state or promote other differentiation pathways such as mesodermal or endodermal, in this case promotes neural fate [36].

*CHIR99021* is a selective inhibitor of glycogen synthase kinase-3 (GSK-3), in this way preventing phosphorylation of β-catenin, which would normally be degraded. Inhibiting β-catenin degradation allows its accumulation in the cytoplasm and subsequent transport into the nucleus, where it can activate target genes that promote neuronal differentiation [38].

*Noggin* is a natural protein that works as an extracellular antagonist of BMP. It binds to BMP molecules before they can interact with their receptors, thereby preventing activation of the BMP pathway, thus promoting differentiation toward neuronal cells [39].

Together, these inhibitors create an environment conducive to the production of dopaminergic neurons by blocking pathways that would otherwise divert differentiation toward other cell types.

#### Immunocytochemistry (ICC) analysis of differentiated cells

To accurately assess the different stages of differentiation using immunocytochemical analysis, it is essential to plate cells on Poly-L-ornithine and Laminin-coated plates (see protocol above). When Matrigel is used as a coating, it can interfere with antibody or fluorescent probe penetration, especially if applied too thickly. Additionally, Matrigel can exhibit autofluorescence, particularly when exposed to light for extended periods. For these reasons, the use of Poly-L-ornithine and Laminin as coating materials is often preferable to Matrigel. Poly-L-ornithine is a cationic polymer that enhances cell adhesion, particularly for neural cell types. Its positive charge interacts with the negatively charged molecules on the cell surface, strengthening the attachment. Laminin, on the other hand, is an extracellular matrix protein that specifically promotes the adhesion and survival of various cell types, including neurons and stem cells. It also supports cell growth and differentiation. By using Poly-L-ornithine and Laminin, the complexity of Matrigel, which contains growth factors and proteins that may interfere with antibody staining, is avoided. Together, Poly-L-ornithine and Laminin provide a more controlled, reproducible substrate suitable for a wide range of cell types. This is particularly advantageous in experiments where maintaining cell morphology and minimizing biological variability are critical, making them ideal for many immunocytochemical assays.

**Fig. 4:**
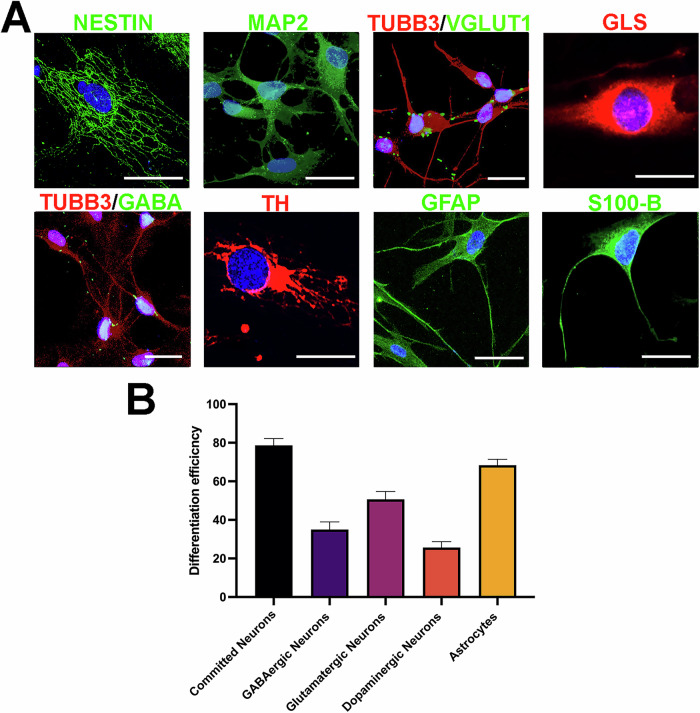
Immunocytochemistry analysis of lineage-specific markers after neural differentiation. **A** Representative picture of ICC staining after neural differentiation. NESTIN was used as Neural Progenitor Markers; MAP2 and TUBB3 were used as a neuron marker; GABA was used as a Gabaergic neuron marker; TH was used as Dopaminergic neuron marker; GLS and VGluT1 were used as Glutamatergic marker; GFAP and S100-B were used as astrocyte markers. The nuclei were counterstained with DAPI (blue). 20X magnification. Scale bar 20 µm. **B** Differentiation efficiency of MUSE cells into various neural cell types. The bar graph illustrates the percentage of MUSE cells that successfully differentiated into committed neurons, GABAergic neurons, glutamatergic neurons, dopaminergic neurons, and astrocytes. Data are presented as mean ± SEM (*n* = 3).

Cells were seeded on coverslips in a 24-well plate for 2 days in the specific related media (NDM, DDM, ADM) and then fixed with 4% formaldehyde for 15 min at room temperature. After fixation, the cells were washed three times with PBS and permeabilized with 0.3% Triton X-100 in PBS for 15 min. Following another PBS wash, the cells were incubated at room temperature for 1 h in a blocking solution containing 0.1% Triton X-100 and 5% bovine serum in PBS. Primary antibodies in blocking solution (as listed in Table 1) were added, and the cells were incubated overnight at 4 °C. All antibodies were used according to the manufacturer’s guidelines. The next day, the cells were washed three times with PBS (5 min each), and then incubated for 1 h at room temperature in the dark with secondary antibodies conjugated to fluorophores. Afterward, the cells underwent three additional 5 min washes with PBS, and the coverslips were mounted. Nuclear staining was performed using DAPI mounting medium, and images were captured using a fluorescence microscope (Leica). The percentage of positive cells was determined by counting at least 500 cells across different microscope fields (Fig. 4).

#### RT-qPCR analysis of differentiated cells

**Fig. 5:**
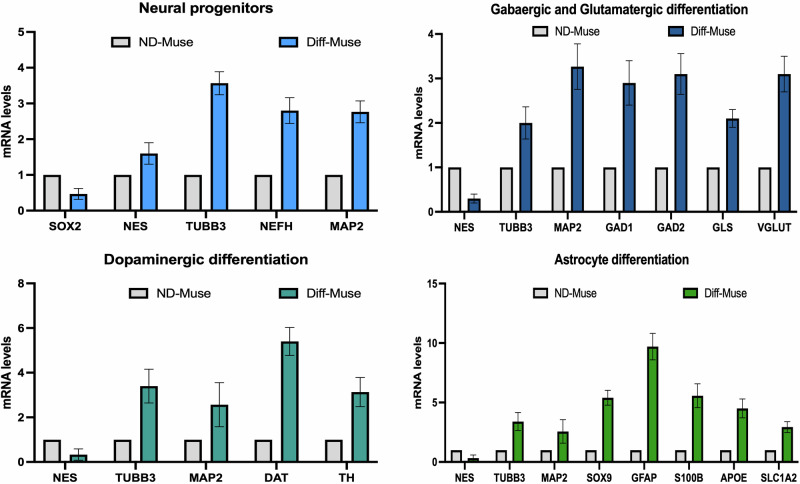
mRNA expression levels of neural differentiation markers. The histograms show the quantitative RT-PCR analysis of neural progenitors’ markers, GABAergic, glutamatergic and dopaminergic markers, astrocytes markers. The mRNA levels were normalized to GAPDH mRNA expression, which was selected as an internal control. Data are expressed as fold changes with standard error (*n* = 3). For each gene, the expression level of not differentiated MUSE (ND-MUSE) is set as the baseline (the arbitrary value is 1).

Total RNA was isolated from cell cultures using the RNeasy Mini Kit (Qiagen), following the protocol provided by the manufacturer. The RNA’s quality and concentration were evaluated using a Nanodrop spectrophotometer. Primer pairs for real-time RT-qPCR reactions were designed based on mRNA sequences retrieved from the Nucleotide Data Bank (National Center for Biotechnology Information) using Primer Express v. 3.0 software (Applied Biosystems). The primer sequences can be found in Table 2. cDNA synthesis was carried out using the 5X ALL-IN-ONE RT MasterMix. GAPDH cDNA regions were used as controls, and the real-time PCR was performed on a Line-Gene 9600 system (Bioer Technology). The reactions followed the manufacturer’s protocol, utilizing BrightGreen 2X qPCR MasterMix. For data analysis, the 2^−ΔΔCT^ method was applied for relative quantification in the real-time PCR experiments (Fig. 5).

## Results

### Isolation and characterization of MUSE cells

We have successfully isolated MUSE cells from stromal cells obtained through skin biopsy (Fig. 1A). When cultured in suspension on poly-HEMA-coated plates, the MUSE cells form clusters, a morphology that is typical for these cells in a non-adherent environment. The suspension culture conditions favor the maintenance of MUSE cells’ stemness. MUSE cells are specifically characterized by the expression of the surface marker SSEA-3. In a representative sample, prior to isolation, only 3.6% of the stromal cells were SSEA-3-positive. However, seven days post-isolation and culture on poly-HEMA, the percentage of cells expressing SSEA-3 rose significantly to 87%. (Fig. 1B). To further validate the stem-like properties of MUSE cells, we performed immunocytochemistry (ICC) analysis to assess the expression of pluripotency markers OCT3/4, SOX-2, and NANOG (Fig. 1C). These markers were positively expressed in MUSE cells, confirming their stem cell identity. Therefore, our results indicate that MUSE cells were successfully isolated and accurately characterized.

### Neural differentiation protocols applied to MUSE cells

MUSE cells were subjected to three neural differentiation protocols: GABAergic and Glutamatergic differentiation, Astrocyte differentiation, and Dopaminergic differentiation. Initially, MUSE cells were induced to form neural progenitors through in vitro culture on Matrigel-coated plates (Fig. 2A) followed by low-binding plates to generate neurospheres (Fig. 2B, C). After a period of proliferation, the neural progenitors (Fig. 2D) were characterized using ICC and RT-qPCR. These progenitors were then differentiated into GABAergic and Glutamatergic neurons, and astrocytes. The MUSE-derived GABAergic and Glutamatergic neurons showed distinct morphology, with GABAergic neurons generally displaying shorter, more branched dendrites, while Glutamatergic neurons exhibited more complex dendritic arbors (Fig. 3A). MUSE cells also differentiated into astrocytes, which showed characteristic star-shaped morphology, typical of mature astrocytes in vitro (Fig. 3B). For dopaminergic differentiation, a protocol specifically directed the progenitors towards a dopaminergic phenotype over a 30-day period. The resulting dopaminergic neurons displayed long, branched dendrites and prominent cell bodies, reflecting typical dopaminergic morphology and function (Fig. 3C). MUSE cells have demonstrated the ability to differentiate into various neural subtypes. Differentiated cells were analyzed for protein expression using ICC and gene expression via RT-qPCR, confirming successful differentiation across protocols.

### Validation of neuronal and astrocytic differentiation through marker analysis

The ICC analysis confirmed the successful differentiation of MUSE cells into both neurons and astrocytes. Specifically, the expression of various markers highlighted the distinct neuronal lineages that the MUSE cells adopted. NESTIN, used as a marker for neural progenitors, was prominently expressed, confirming the presence of neural progenitors. MAP2 and TUBB3, markers for committed neurons, were also detected, indicating successful neuronal differentiation. Furthermore, the GABAergic and dopaminergic differentiation was verified by the expression of GABA and TH markers, respectively. Additionally, GLS and VGLUT1 confirmed the presence of glutamatergic neurons. Astrocytic differentiation was also evident, as indicated by the expression of GFAP and S100-B, which are well-established markers of astrocytes (Fig. 4A). These findings demonstrate the ability of MUSE cells to differentiate into specific neuronal and astrocytic subtypes.

To quantify the efficiency of differentiation from MUSE cells to various neural subtypes, we systematically analyzed the proportion of cells expressing specific neuronal and glial markers after the differentiation protocol. Using immunocytochemistry, we were able to determine the following differentiation efficiencies (Fig. 4B): approximately 78% of the differentiated cells expressed markers consistent with committed neurons. Of these, the distribution into specific neuronal subtypes was as follows: GABAergic neurons: 34% of the neuronal population expressed GABA. Glutamatergic neurons: 49% expressed VGLUT1 and GLS. Dopaminergic neurons: 26% exhibited expression of TH, confirming dopaminergic phenotype. After astrocyte differentiation, approximately 68% of the differentiated cells were identified as astrocytes, expressing GFAP or S100B as markers for astrocytic cells. These data suggest a robust ability of MUSE cells to differentiate into multiple neural lineages, with a significant proportion achieving a mature and specific neuronal or glial phenotype. The successful differentiation into these subtypes highlights the potential of MUSE cells as a versatile tool for modeling neurological diseases and testing therapeutic interventions.

### Gene expression analysis of differentiated MUSE cells

The RT-qPCR analysis further confirmed the differentiation potential of MUSE cells into distinct neural lineages, as evidenced by the overexpression of specific markers. For neural progenitors, significant upregulation of *TUBB3*, *NEFH*, and *MAP2* was observed in differentiated MUSE cells, indicating their progression toward neuronal lineage, with *NES* also elevated but to a lesser extent. In terms of GABAergic and glutamatergic differentiation, key markers such as *GAD1*, *GAD2* (Gabaergic neuron markers) and *GLS* and *VGLUT1* (Glutamatergic neuron markers) showed enhanced expression, confirming the differentiation into GABAergic and glutamatergic neurons. Dopaminergic differentiation was supported by the marked increase in *DAT* and *TH* expression. Moreover, astrocytic differentiation was demonstrated by the significant overexpression of *GFAP* and *S100-B*, with other astrocytic markers such as *SOX9* and *APOE* also elevated in differentiated MUSE cells (Fig. 5). These results collectively reinforce the multipotent nature of MUSE cells and their ability to differentiate into multiple neural subtypes, further validating the effectiveness of neural differentiation protocols.

## Discussion

Research focusing on neurological and psychiatric conditions often highlights deficits in mature neuron functionality within in vitro environments, yet such studies inherently encounter significant limitations. Notably, the critical process of neurogenesis is frequently neglected in these studies, despite growing evidence linking disruptions in neural stem cell processes—like faulty cell differentiation, biased lineage choices, and accelerated aging or death of cells—to the pathogenesis of these diseases. As a result, there is a compelling need for in vitro models to evolve, ensuring they encompass both the complex interdependencies of neural networks and the foundational developmental changes that predispose to neurological and psychiatric ailments.

Neural progenitor cells (NPCs) possess unique functional characteristics that are crucial for modeling neurological diseases. Unlike fully differentiated neurons, NPCs exhibit high plasticity and proliferative capacity, which are pivotal for studying disease mechanisms and potential therapeutic interventions. These features allow NPCs to model the dynamic processes of neurodevelopment and degeneration, providing insights into the early stages of disease formation and progression. In contrast, while fully differentiated neurons offer a snapshot of mature cellular function, they lack the developmental context and regenerative potential inherent to NPCs, which is often critical for understanding and intervening in disease pathways. The use of NPC is particularly pertinent for studying psychiatric disorders like mood disorders, and neurodegenerative diseases that exhibit early progenitor cell dysfunction. For instance, research has demonstrated that in schizophrenia, NPCs show abnormal patterns of differentiation and migration, which may contribute to the pathophysiology of the disorder [40]. Similarly, in bipolar disorder, NPCs have been found to exhibit altered neurogenesis, which is thought to affect brain structure and function, potentially underlying mood instability [41]. In the context of neurodegenerative diseases, such as Alzheimer’s disease, NPCs have shown impaired proliferative abilities and reduced capacity to differentiate into necessary neuronal subtypes, which are critical for maintaining cognitive functions [42]. In Parkinson’s disease, deficits in NPC proliferation and differentiation lead to a reduction in dopaminergic neurons, directly contributing to the neurological deficits observed in this condition [43]. These studies underscore the relevance of NPCs in understanding the cellular and molecular foundations of such disorders, supporting our focus on these cells to model disease mechanisms more accurately.

We have developed specialized protocols for differentiating MUSE cells into neural progenitors, subsequently focusing on the lineage specification of these progenitors into GABAergic, glutamatergic, dopaminergic neurons, and astrocytes. This advancement provides a refined in vitro model that is particularly effective for probing the cellular mechanisms implicated in neurodegenerative and psychiatric disorders, including schizophrenia, bipolar disorder, and Alzheimer’s disease. Unlike iPSC-based models, which are often hampered by genetic variability and epigenetic memory [44, 45], MUSE cells offer a more stable and physiologically relevant platform. These cells not only mirror critical aspects of neuronal and glial dysfunction integral to these conditions but also are readily obtainable from patients, enhancing their practicality for clinical research. The detailed assessment of differentiation efficiencies into key neural subtypes enables precise evaluation of the model’s fidelity and its applicability to study specific neurological phenomena. The significant proportion of MUSE cells that successfully differentiate into both neuronal and glial phenotypes underscores their utility for comprehensive studies into the origins and development of neurodevelopmental, neurodegenerative, and psychiatric disorders.

While the differentiation protocols successfully generated committed neurons or astrocytes capable of expressing differentiated cell markers, further evaluation of their functional properties is needed.

Many studies on neural diseases focus on the in vitro electrophysiological activity of neurons derived from the differentiation of progenitor cells. However, we did not adopt this experimental approach, as it is essential to recognize the inherent limitations of these in vitro models [46]. Studies have shown that in vitro electrophysiology can introduce artifacts that do not accurately reflect real physiological conditions. For instance, the absence of a three-dimensional environment and the lack of cell-cell interactions found in vivo can significantly alter the electrophysiological properties observed in culture [47]. Additionally, the long-term culture necessary for developing electrophysiologically mature neurons can affect the stability and reproducibility of the results [48].

Given these considerations, we focused on developing and characterizing a robust protocol for differentiating MUSE cells into various neuronal subtypes, aiming to optimize the cellular models for future studies that may integrate more complex assays, including 3D cultures and potentially in vivo studies. The utility of these models in 3D organoid could provide a more comprehensive and physiologically relevant platform for studying cell-cell interactions and disease mechanisms. In vivo studies allow for the observation of neuronal activity within intact neural circuits and under real physiological conditions, providing data that are more representative of brain function. This approach can overcome the limitations and artifacts associated with in vitro models, offering a more robust evaluation even of their electrophysiological characteristics [48].

In our opinion, the in vitro model alone may be more suitable for studies on stem cell properties and the lineage specification of neural cells, given the causative role that any impairment of these processes may play in the onset of neurological diseases.

## Conclusion

The establishment of robust protocols for generating neural and glial cells facilitates a more accurate study of neurotransmitter system dysfunctions and astrocyte alterations, leading to potential new therapeutic targets. Moreover, our protocol enables the study of neural progenitor cell dysfunctions, providing an essential platform for investigating diseases characterized by impaired progenitor cell behavior and offering insights into their role in neurodevelopmental and neurodegenerative pathologies. These advanced in vitro models can also reduce reliance on animal models in early-stage research, offering a more ethical and potentially cost-effective alternative for drug discovery and therapeutic development.
